# Ranibizumab injection and laser photocoagulation to treat type 1 retinopathy of prematurity after 40 weeks post menstrual age: a retrospective case series study

**DOI:** 10.1186/s12886-019-1067-4

**Published:** 2019-02-26

**Authors:** Jiao Lyu, Qi Zhang, Chunli Chen, Yu Xu, Xunda Ji, Peiquan Zhao

**Affiliations:** 1 Department of Ophthalmology, Xinhua Hospital, Medical School, Shanghai Jiao Tong University, 1665 Kong Jiang Road, Shanghai, 200092 China; 2 Department of Ophthalmology, Shengli Oilfield Central Hospital, 31 Jinan Road, Dongying, 257000 Shan Dong Province China

**Keywords:** Retinopathy of prematurity, Older ROP, Treatment, Vascular endothelial growth factor, Laser, Postmenstrual age

## Abstract

**Background:**

Type 1 retinopathy of prematurity (ROP) is occasionally observed in preterm infants after the postmenstrual age (PMA) of 40 weeks; however, evidence-based treatment guidelines are largely lacking. In this study, we report the clinical characteristics of preterm infants with type 1 ROP at PMA of > 40 weeks and compare the treatment outcomes of intravitreal ranibizumab (IVR) and laser therapy.

**Methods:**

Twenty-seven eyes of 14 infants, primarily treated for type 1 ROP after 40 weeks PMA by IVR (17 eyes in 9 infants) or by laser photocoagulation (10 eyes in 5 infants) were included in this retrospective analysis. The preoperative fundus characteristics and the structural outcomes and additional treatment after 6 months were analyzed.

**Results:**

Of the 27 eyes, 20 eyes (74%) had zone II stage 3 plus disease (+) ROP and 7 eyes had zone II stage 2 + ROP. Seventeen (63%) eyes showed thick fibrous ridges. After primary treatment at 40–48 weeks PMA, ROP regression was observed in a similar proportion of eyes in the IVR and laser groups (88% vs. 70%; *p =* 0.326); complete vascularization was observed in 24% eyes in the IVR group. Compared to laser group, a higher proportion of eyes in IVR group received additional treatment (IVR group 76% vs. laser group 30%; *p =* 0.040), for unresolved peripheral avascularity in 11 eyes and ROP progression with fibrotic contraction in 2 eyes after primary IVR.

**Conclusion:**

Preterm infants with type 1 ROP at > 40 weeks PMA displayed enhanced fibrotic proliferation. Both primary IVR and laser effectively promote ROP regression. Primary IVR cannot guarantee full retinal vascularization but is associated with a risk of fibrotic contraction.

## Background

Retinopathy of prematurity (ROP) is a vaso-proliferative disease in which the timing of retinal vascular events correlates closely with postmenstrual age (PMA) [1]. The onset of acute-phase ROP peaks at the PMA of approximately 35–39 weeks and ROP involution mostly occurs before the PMA of 44 weeks [2–4]. Timely management of ROP can optimize treatment efficacy and minimize the risk of progression to retinal detachment. Occasionally, preterm infants with type 1 ROP after 40 weeks of PMA are encountered in clinical settings, possibly due to late onset of ROP or delayed screening [5, 6]. In the Cryotherapy for Retinopathy of Prematurity (CRYO-ROP) study, 5% of infants manifested acute-phase ROP disease after 41.5 weeks and 1% after 46.3 weeks [2, 3]. Although uncommon, type 1 ROP at an advanced PMA requires meticulous management, considering the undetermined natural course and the lack of relevant clinical guidelines [6].

Anti-vascular endothelial growth factor (VEGF) therapy and laser ablation have been shown to be effective in the treatment of type 1 ROP [7, 8]. Compared to laser photocoagulation, anti-VEGF therapy may promote rapid regression of ROP, allow for retinal vascularization, and risk retreatment because of recurrent neovascularization, persistent peripheral avascularity, or fibrotic proliferation [7–10]. Most studies have investigated outcomes of primary treatment of type 1 ROP before PMA of 40 weeks; however, data pertaining to anti-VEGF and laser treatment at an older age are relatively lacking and scattered [6–9, 11–13]. In particular, the treatment efficacy and anatomical outcomes of primary anti-VEGF therapy for treatment of type 1 ROP at an advanced PMA are not clear. Herein, we describe a series of cases with type 1 ROP after PMA of 40 weeks and assess the structural outcomes associated with the two treatments: primary intravitreal ranibizumab injection (IVR) and primary laser photocoagulation.

## Methods

All patients in this retrospective case series were treated at the ophthalmic center at the Xinhua hospital, School of Medicine, Shanghai Jiao Tong University, China. Study protocols were in accordance with the tenets of Declaration of Helsinki and approved by the hospital ethics committee. Written informed consent for off-label IVR treatment for ROP was obtained from legal guardians of all patients after detailed counseling regarding the potential benefits (possibly, quick regression after single injection, anterior progression of retinal vascularization, preservation of peripheral visual field) and risks (possibly, infection, hemorrhage, fibrosis, retreatment) of the treatment. The study and off-label use of Ranibizumab were certified by Ethics Committee of Xin hua Hospital and informed consent to participate was exempted due to retrospective nature of the study (Approval No. XHEC-D-2018-028).

The Xinhua hospital has a major referral neonatal intensive care unit for premature infants with varying levels of ROP. Medical records of consecutive patients admitted for ROP screening between January 2012 and June 2016 were reviewed. Exclusion criteria were presence of other associated pediatric retinopathies and suspected familial exudative vitreoretinopathy in preterm infants who showed a disease course not consistent with ROP or had a family history. A total of 1694 records with a diagnosis of ROP were identified. Thirty-three eyes of 17 infants received primary treatment for type 1 ROP after PMA of 40 weeks and 16 of these were followed up for > 6 months. Two infants who were diagnosed with stage 4 or 5 ROP in one eye before treatment were excluded, as they were scheduled for a more aggressive treatment plan to monitor the lateral eye with type 1 ROP. Thus, data pertaining to a total of 27 eyes of 14 infants were included in the analysis.

The screening guidelines for ROP in China are as follows: (1) gestational age (GA) ≤34 weeks; (2) birth weight (BW) ≤2000 g; and (3) preterm infants who had been ventilated for at least 1 week or who received supplemental oxygen for > 30 days. ROP was diagnosed and classified according to the International Classification of ROP [14]. Type 1 ROP was defined as any stage of ROP (+) or stage 3 ROP in zone I, or stage 2 or 3 ROP (+) in zone II, based on the Early Treatment for Retinopathy of Prematurity Randomized Trial [15].

Fundal examination was performed and wide-angle retinal images were captured using RetCam (Clarity Medical Systems, Pleasanton, CA, USA) in serial examinations. All fundus images were reviewed by two retinal specialists to render a consensus. Binocular indirect ophthalmoscopy with scleral indentation was performed as needed. B-ultra scan was performed if retinal detachment was suspected.

### Treatment groups and follow-up

Twenty-seven eyes of 14 patients were subdivided into two groups according to the primary treatment: (1) IVR group (received primary IVR) (2) laser group (received primary laser photocoagulation). Before June 2013, primary laser photocoagulation was administered routinely for patients with type 1 ROP after the PMA of 40 weeks. With the growing role of anti-VEGF therapy in ROP, we began to administer primary IVR for some patients with type 1 ROP in more posterior zones at the PMA of > 40 weeks.

Primary treatment was administered within 24 h of diagnosis under topical anesthesia with 0.5% proparacaine (Alcaine, Alcon Laboratories Inc., USA). Laser photocoagulation was performed using 810 nm diode laser (IRIS Medical Oculight SL 810 nm infrared laser; Iris Medical Inc., USA). Confluent laser burns were applied to the entire avascular retina. The laser settings used were: energy 200–350 mW; repeat mode with 0.2 s exposure time; interval time: 0.2 s. The procedure for IVR was as follows: use of sterile gloves; insertion of a lid speculum; instillation of topical povidone-iodine; injection of ranibizumab (0.25 mg/25 μL) using a sterile 30-gauge needle at 0.5–1 mm posterior to the limbus; removal of the needle with simultaneous compression using a sterile cotton tip; instillation of topical tobramycin; and removal of the speculum. Both IVR and laser therapy were performed by the same surgeon. Follow-up was scheduled at days 3 and 7 after treatment and then weekly or biweekly or monthly until complete involution of ROP with vascularization of zone III, but not necessarily with vessels reaching the temporal ora seratta.

Additional treatment by laser photocoagulation was applied to avascular retina or skipped areas in the following conditions: (1) ROP persistence: persistence of neovascularization or plus disease two weeks after primary treatment; (2) ROP reactivation: return of neovascularization or plus disease after initial ROP regression; (3) ROP progression: progression to a more advanced stage; and (4) unresolved peripheral avascularity of zone II or III after primary IVR. Vitrectomy was performed in advanced stages of ROP when removal of epi-retinal tractional fibrosis was indicated. An unfavorable anatomical outcome was defined as the presence of at least one of the following findings: temporally dragged retina over the nerve, macular ectopia, retinal fold, or retinal detachment.

### Outcome measurements and statistical analysis

Baseline demographic characteristics and Retcam photographs of serial fundal examinations were evaluated. The proportion of eyes with ROP regression, additional treatment, and unfavorable anatomy were compared between the two groups 6 months after primary treatment as an endpoint. Statistical analyses were performed using SPSS 22.0 Windows (SPSS, Inc., Chicago, IL, USA). A *p* value of < 0.05 (2-sided) was considered significant for all tests. Data are presented as mean ± SD for continuous variables or as frequency (percentage) for categorical variables. Between-group differences were assessed using Chi-squared test or Fisher’s exact test for categorical variables, and Student *t*-test or Mann–Whitney test on ranks for continuous variables depending on the distribution. All *p*-values are two-tailed and considered significant if < 0.05.

## Results

### Characteristics

Twenty-seven eyes of 14 patients with type 1 ROP treated at 41.4 ± 2.2 weeks PMA (range, 40–48 weeks) were analyzed in this study. One infant showed avascular zone II at the first fundus screening test before the PMA of 40 weeks, while 13 infants had their first screening for ROP after the PMA of 40 weeks due to lack of awareness of the standard screening schedule. The demographic and fundus characteristics are shown in Table 1.

**Table 1:** Characteristics of infants with Type 1 ROP after the post-menstrual age of 40 weeks

Variables	All infants	Comparison between Group 1 and 2		
		Group 1	Group 2	*p* value
		(Primary IVR)	(Primary laser)	
Patients, n	14	9	5	/
Male, n (%)	8	7 (78%)	0 (0%)	0.021* (Fisher’s exact test)
GA, weeks (mean ± SD)	28.9 ± 2.3	29.0 ± 2.2	28.8 ± 2.6	0.882 (Student *t*-test)
(range)	(26.0–33.0)	(27.0–33.0)	(26.0–32.0)	
BW, grams (mean ± SD)	1227.1 ± 347.9	1200.5 ± 360.3	1275.0 ± 359.6	0.717 (Student *t*-test)
(range)	(815–2000)	(815–2000)	(980–1800)	
PMA at diagnosis, weeks [mean ± SD (range)]	41.4 ± 2.2	40.8 ± 1.0 (40–43)	42.6 ± 3.4 (40–48)	0.518 (Mann-Whitney test)
Follow-up, months [mean ± SD (range)]	12.5 ± 6.9 (6–24)	11.0 ± 5.9 (6–24)	15.2 ± 8.3 (6–24)	0.290 (Student *t*-test)
Eyes, n	27	17	10	
Zone 2 stage 3 with plus, n (%)	20	14 (82%)	6 (60%)	0.365 (Pearson’s Chi-squared test)
Zone 2 stage 2 with plus, n (%)	7	3 (18%)	4 (40%)	/
Posterior zone 2 disease		12 (86%)	0 (0)	< 0.001(Fisher’s exact test)
Fibrotic ridges, n (%)	17	12 (70%)	5 (50%)	0.415
Hemorrhage on fibrovascular ridge, n, (%)	11	10 (59%)	1 (10%)	0.018* (Fisher’s exact test)

*BW* birth weight, *DD* disc diameter, *GA* gestational age, *IVR* intravitreal injection of ranibizumab, *PMA* postmenstrual age, *ROP* retinopathy of prematurity, *SD* standard deviation

*significant between-group difference

The IVR group comprised of 9 patients (17 eyes), while the laser group comprised of 5 patients (10 eyes). Of the 27 eyes, 20 eyes (74%) had preexisting zone II stage 3 + ROP and the remaining 7 eyes (26%) had zone II stage 2+ ROP. As for plus disease, 18 eyes (67%) showed highly vascular activity in all four quadrants while 9 eyes (33%) showed relatively mild vascular dilation and tortuosity. A significant between-group difference was observed with respect to the ratio of posterior zone II involvement (*p* < 0.001, Fisher’s exact test) and sex (*p* = 0.021, Fisher’s exact test); however, there was no significant difference with respect to the mean BW, GA, or PMA at the time of diagnosis and treatment.

**Fig. 1:**
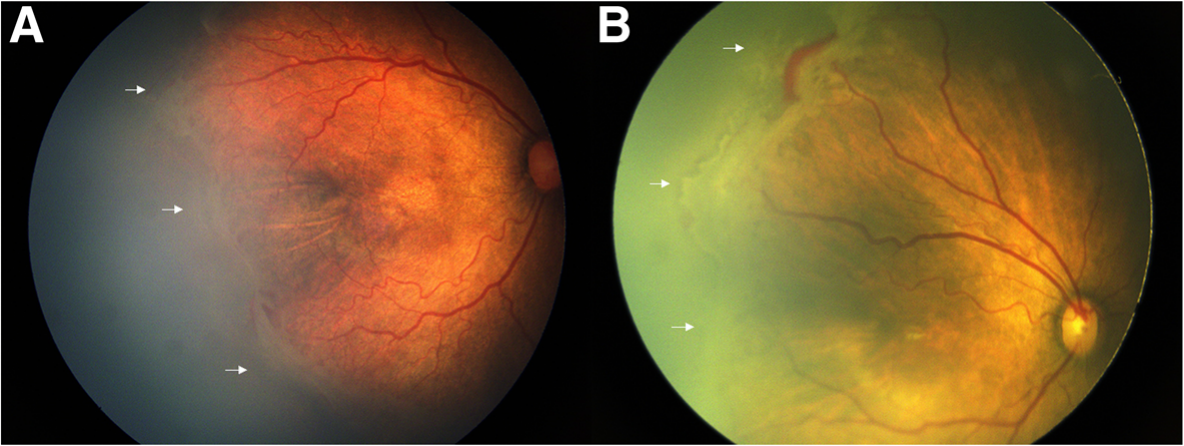
Fundus images (**a**) and (**b**) of Type 1 ROP in posterior zone II after 40 weeks PMA show thick and paralleled fibrovascular ridges that hinder vascularization and pose tractional force. **b** shows relatively moderate vascular activity and straightening of peripheral vessels. White arrows point to these ridges at the vascular endings

Before treatment, 17 of the 27 eyes (63%) showed thick, whitish, well demarcated, and paralleled fibrovascular ridges where the vessels stopped advancing from the initial ridge to the more anterior ridge. Additionally, eleven (41%) eyes exhibited hemorrhage on ridge or in the vitreous cavity (Fig. 1), and 10 of the 11 eyes were in the IVR group.

### Anatomical outcomes

Anatomical outcomes at 6 months after primary treatment in the two groups are presented in Table 2. After primary treatment, the proportion of eyes that exhibited regression of type 1 ROP was comparable in the two groups (88% vs. 70%; *p* = 0.326, Fisher’s exact test). In the IVR group, 7 (70%) of 17 eyes had anterior retinal vascularization and 4 eyes eventually exhibited zone III vascularization.

**Table 2:** Anatomical outcomes of infants with type 1 ROP in the two groups within 6 months after primary treatment

Variables	Group 1 (Primary IVR)	Group 2 (Primary laser)	*p* value
Eyes,	17	10	
Regression of acute ROP, n (%)	15 (88%)	7 (70%)	0.326 (Fisher’s exact test)
Additional treatment, n (%)	13 (76%)	3 (30%)	0.040*(Fisher’s exact test)
Causes			
*ROP persistence*, n	0	3	
*ROP reactivation*, n	0	0	
*ROP progression*, n	2	0	
*Avascularity in zone II or zone III*	11	0	
Methods			
*Laser*, n	13	3	
*IVR*, n	0	0	
*Vitreoretinal surgery*, n	1	0	
Unfavorable outcomes, n (%)	2 (12%)	0 (0%)	0.516 (Fisher’s exact test)
*Temporally dragged retina over the nerve*, n	2	0	
*Retinal detachment or fold*, n	0	0	

*GA* gestational age, *IVR* intravitreal injection of ranibizumab, *ROP* retinopathy of prematurity

*significant between-group difference

**Fig. 2:**
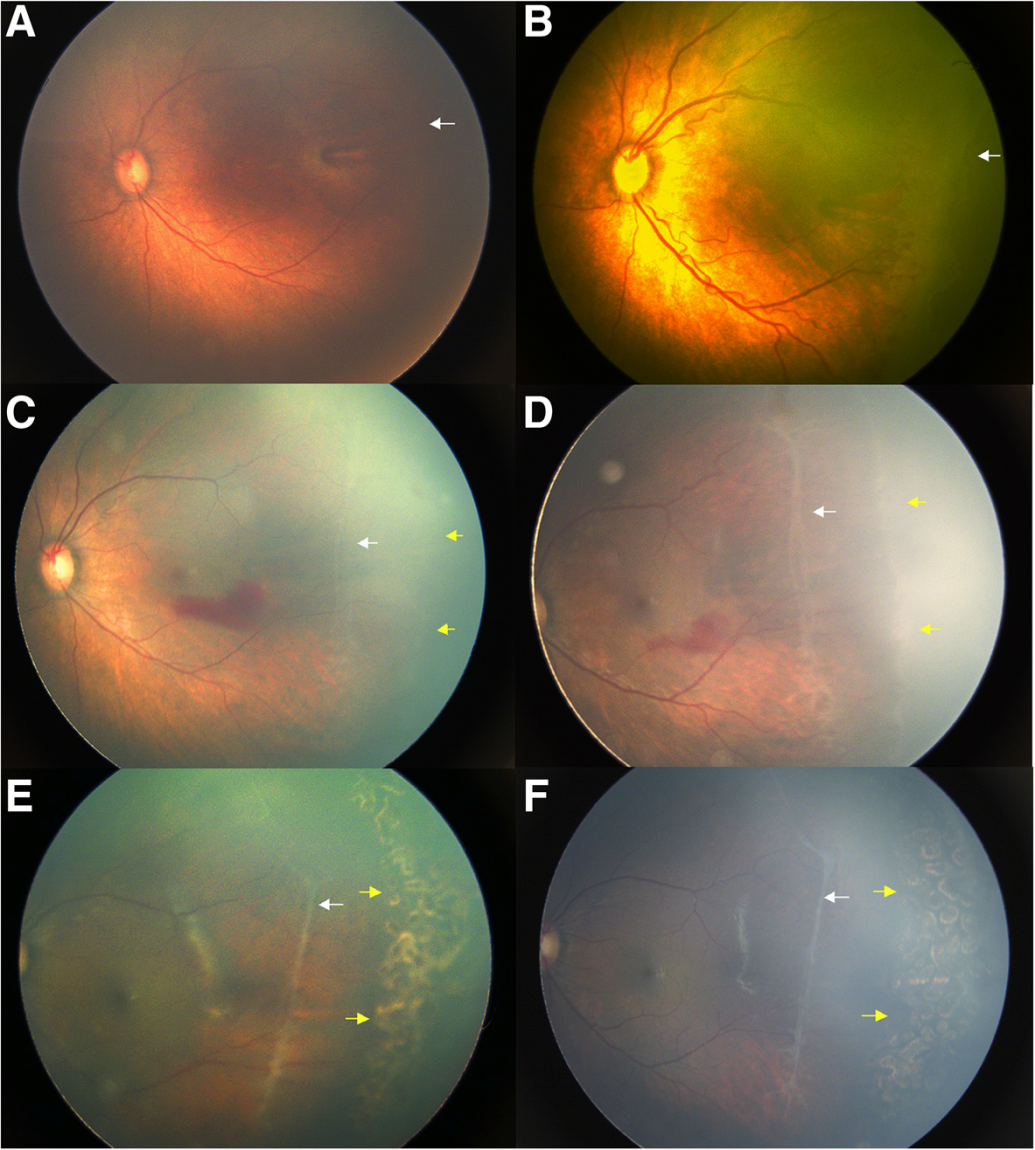
Fundus images of an infant with Type 1 ROP who received primary IVR treatment. White arrows point to the original edge of vascular retina in temporal periphery and yellow arrows indicate the edge of vascular retina after treatment. **a** The left eye shows vascularization of zone II posterior at 37 PMA weeks; (**b**) The eye developed zone II stage 3 with plus disease at 41 weeks PMA and received primary IVR; (**c**) Regression of ROP and retinal vascularization are observed at 45 weeks PMA; (**d**) Persistence of peripheral avascularity with increased activity of peripheral vessels at 48 weeks PMA indicated further treatment; straightening of peripheral vessels is prominent. **e** Laser photocoagulation was applied to the peripherally-retrograding avascular zone. The anterior extent of retinal vascularization was preserved; (**f**) The eye showed ROP involution at 66 PMA weeks

However, additional treatment was performed in a greater proportion of eyes in IVR group than in the laser group (76% vs. 30%; *p =* 0.040, Fisher’s exact test). After completion of all treatment, unfavorable outcomes were observed in 2 eyes in the IVR group as against none in the laser group; however, the between-group difference in this respect was not statistically significant. In the IVR group, 11 eyes from 6 infants showed avascularity in zone II or III 4.1 ± 2.1 (range, 1.5–7) weeks after primary IVR and then received laser ablation (Fig. 2). One infant with stage 3+ disease had reduced vascular activity, but developed 4a ROP and vitreous hemorrhage in both eyes due to fibrotic contraction after primary IVR. The infant then received several sessions of laser in both eyes and underwent vitrectomy in the right eye to remove the hemorrhage and reattach the retina. In the laser group, 3 eyes showed persistent type 1 ROP 2 weeks after primary laser and then received additional laser to the skipped areas. At the last visit, 2 eyes with ROP progression in the IVR group showed temporally dragged retina over the nerve. No other unfavorable outcomes were observed in the treated eyes.

## Discussion

This study reported the characteristics and treatment outcomes for preterm infants with type 1 ROP after the PMA of 40 weeks. Infants with type 1 ROP after 40 weeks PMA tended to show enhanced fibrotic proliferation. Primary IVR and laser photocoagulation were effective in inducing ROP regression; however, primary IVR may risk unresolved peripheral avascularity and fibrotic contraction.

In this study, a large proportion of eyes with type 1 ROP showed aberrant vascular activity and increased fibrotic proliferation after the PMA of 40 weeks. It is likely that the persistent large area of avascular retina and persistent vascular activity are responsible for the progression to type 1 ROP at this late time period. Based on the observation, the ridges in type 1 ROP at an advanced age appear thicker, wider, flatter, and whitish than those at an early age, while the latter appear more reddish and exhibit a more blurred edge. Furthermore, the vascular activity seems to decrease as PMA exceeds 40 weeks. We speculate that the characteristics observed in our cases may reflect a specific status relevant to advanced PMA when there is a shift from acute vaso-proliferation to fibrotic involution of ROP. These morphological changes documented in previous reports also support this speculation. In a study by Ho et al [6], 18 eyes (9 patients) with ROP received treatment after the PMA of 45 weeks; typical fundus images of 4 eyes revealed prominent fibrotic ridges and dragging vascular endings. In a case-series report by Gupta et al [13], 10 of 13 eyes were treated for ROP milder than type 1 after the PMA of 40 weeks and showed retinal dragging by fibrotic components and vitreous hemorrhage. Knowledge of the morphological characteristics will facilitate our understanding and provide insights for monitoring of type 1 ROP at an advanced PMA.

This study confirmed the effectiveness of anti-VEGF therapy and laser ablation in promoting regression of neovascularization and plus disease of type 1 ROP at an advanced PMA. ROP is a biphasic disease: phase 1 (22–30 weeks) with delayed physiologic retinal vascular development due to relative hyperoxia and decreased levels of VEGF; phase 2 (31–44 week PMA) with vaso-proliferation owing to relative hypoxia and increase of VEGF [16, 17]. Direct suppression of the VEGF concentration in the vitreous by anti-VEGF drugs at the PMA of 40–43 weeks rapidly reduced aberrant vascular activity and promoted neovascularization regression to some extent, followed by anterior vascularization in some cases. Thus, greater preservation of retina may serve as a reasonable ground for primary IVR in case of posteriorly located disease, as shown in our series. Compared with primary IVR, primary laser sometimes yields a delayed vascular response, as observed in 3 eyes which showed ROP persistence after laser therapy. This is likely attributable to the fact that laser indirectly reduces intraocular VEGF level by ablating avascular zone and reducing oxygen requirement [8].

It is noteworthy that peripheral avascularity and fibrosis after primary IVR at an advanced PMA is a major indication for further ROP management. No ROP recurrence was observed in the study, which is different from previous studies of infants treated at an earlier PMA that showed a substantial rate of ROP recurrence after single IVR [18, 19]. Since physiologic vascularization may slow down at an advanced PMA, it is reasonable to anticipate less recurrent neovascularization, but more peripheral avascularity and fibrogenesis after a single anti-VEGF therapy [6]. Another factor may be the relatively short observation time after IVR, as prophylactic laser treatment was done in some cases. Laser ablation of avascular zone is done in advance based on the following considerations. On one hand, anti-VEGF therapy allows anterior progression of retinal vascular edge, but full retinal vascularization may not necessarily be achieved [20, 21]. Chronic ischemia associated with peripheral avascularity calls for active management to prevent recurrence of neovascularization and attendant vitreoretinopathies [6, 13, 21, 22]. On the other hand, increased fibrosis after anti-VEGF therapy has been a concern in ROP management [23]. Occasionally, retinal traction or “crunching” of retina can occur rapidly after primary IVR, if there is no RPE-retina adherence in the avascular zone induced by initial laser ablation [24]. In this perspective, timely laser ablation is a requisite for IVR-treated eyes with increased fibrotic proliferation at an advanced PMA when cicatrices may persist.

Compared to anti-VEGF therapy, laser ablation reportedly can also lead to fibrotic contraction and retinal detachment, although no progression of ROP after laser was observed in our patients after vigorous management [25]. In the ET-ROP study, 63 patients (89 eyes) out of the 401 patients who received laser therapy developed retinal detachment [25]. In eyes with active vascularization, laser ablation can lead to excessive inflammation after breakdown of the blood-retinal barrier, which induces fibrosis and cicatrices [8]. Thus, to minimize unfavorable outcomes in severe ROP at advanced PMA, we advocate a combination therapy by IVR and laser ablation to promote rapid regression of neovascularization, as well as to induce RPE-retina adhesion.

Several limitations should be considered during interpretation of our findings. This was a retrospective study and the precise timing of vascular events in ROP infants cannot be determined. Limited cases in a single institution may not represent all patterns of ROP management at an advanced PMA. The characteristics of ROP in this sample of Chinese patients are different from those typically found in developed countries. In addition, our findings may not be universally applicable, mainly due to the differences in the health care systems. Moreover, there may be an element of bias induced by the differences between the two groups with respect to the sex ratio of infants and the proportion of affected eyes with posterior zone II involvement and pre-existing hemorrhage. Although the mechanism is not well established, male preterm infants were shown to be associated with a higher risk of developing referral-warranted ROP as well as severe visual impairment [26, 27]. The difference of the two baseline factors possibly reflected the tendency of administering primary IVR in more severely affected eyes in the real-world condition. Additionally, the historical time for treatment in the two groups was not consistent, which also can lead to bias. Besides, FA was not performed to define the fundus characteristics of treated eyes or to aid therapeutic decisions for additional treatment.

## Conclusions

Despite these limitations, the study provided first hand data regarding anti-VEGF and laser treatment in older PMA infants with type 1 ROP. In conclusion, preterm infants with type 1 ROP after the PMA of 40 weeks may display unique fundus characteristics which may affect the treatment algorithm. Primary IVR results in rapid ROP regression and allows retinal vascularization, but may require additional management for peripheral avascularity and risks fibrosis. A multicenter study with a large sample size may help to further evaluate the anatomical outcomes associated with ROP treatment at an advanced PMA.

## Abbreviations

FEVR: Familial exudative vitreoretinopathy
IVR: Intravitreal ranibizumab
PMA: Postmenstrual age
ROP: Retinopathy of prematurity
VEGF: Vascular endothelial growth factor
